# Trust and Treatment: Overcoming Post‐Pandemic Anxiety in Healthcare

**DOI:** 10.1002/hsr2.71220

**Published:** 2025-09-09

**Authors:** John Patrick C. Toledo

**Affiliations:** ^1^ Department of Theology and Religious Education De La Salle University Manila Philippines

## Transparency Statement

I, John Patrick C. Toledo, the sole author of the article, affirm that this manuscript is an honest, accurate, and transparent account of the study being reported; that no important aspects of the study have been omitted; and that any discrepancies from the study as planned (and, if relevant, registered) have been explained.

Dear Editor,

I have read with great interest the study entitled “Relationship Between Fear of Coronavirus Disease 2019 and Patient Preferences for Medical Services in Ghana: A Cross‐Sectional Study” by Mensah and colleagues. The relationship between patients' fear of COVID‐19 and patients' concerns about healthcare access [1]. Delays in diagnosis, lower health outcomes, and an overworked healthcare system can result from this phenomenon. Given that the anxiety dynamics seen during health emergencies are probably universal, the findings from Ghana are extremely relevant to other nations, especially those with comparable socioeconomic circumstances. In order to create efficient communication plans and actions, public health officials must have a thorough understanding of these patient preferences. One important lesson learned is how crucial it is to increase public confidence and knowledge about the effectiveness and security of medical settings. Fear may subside and prudent use of medical services may be encouraged when people believe that hospitals are secure and well‐prepared.

Additionally, the study subtly emphasizes the necessity of psychological therapies to lessen pandemic‐related worry and panic. Organizing systems for psychologically informed interventions [2] is important. This could entail destigmatizing medical visits during outbreaks, promoting hospital safety procedures in public awareness campaigns, and providing mental health support services. In order to guarantee ongoing and fair access to medical care, treating the psychological aspects of health crises is ultimately just as important as treating the physical illness itself.

## Author Contributions

**John Patrick C. Toledo:** conceptualization, writing – original draft.

## Ethics Statement

All procedures were conducted in strict adherence to ethical regulations.

## Conflicts of Interest

The author declares no conflicts of interest.

## Supporting information

Authoranddapos_s_Statement.

## Data Availability

Data sharing is not applicable to this article as no data sets were generated or analyzed during the current study.
