# Supplementary material for: Trust and Treatment: Overcoming Post‐Pandemic Anxiety in Healthcare
Source: Health Sci Rep. 2025 Sep 9;8(9):e71220. doi: 10.1002/hsr2.71220 (PMC12420359; doi:10.1002/hsr2.71220)
Supplement: Supplementary file 1 — Authoranddapos_s_Statement. [file HSR2-8-e71220-s001.docx]

### Funding

The author(s) declare that no funding sources or financial relationships influenced the study design; collection, analysis, and interpretation of data; writing of the report; or the decision to submit the report for publication.

### Author Contributions and Transparency

I, John Patrick Toledo, the sole author of the article, affirm that this manuscript is an honest, accurate, and transparent account of the study being reported; that no important aspects of the study have been omitted; and that any discrepancies from the study as planned (and, if relevant, registered) have been explained.

Conflict of Interest Statement

The author declares no conflict of interest. All procedures were conducted in strict adherence to ethical regulations.
